# Targeting KRAS in Cancer Therapy: Beyond Inhibitors

**DOI:** 10.1002/mco2.70267

**Published:** 2025-06-25

**Authors:** Chunhui Chen, Zhiqiang Peng, Lingqiang Zhang

**Affiliations:** ^1^ Department of Gastroenterology The Second Affiliated Hospital of Guangzhou University of Chinese Medicine Guangzhou China; ^2^ State Key Laboratory of Medical Proteomics, National Center for Protein Sciences (Beijing) Beijing Institute of Lifeomics Beijing China

1

A recent study by Popow et al. published in *Science* described a heterobifunctional small‐molecule pan‐KRAS degrader ACBI3 [1], while two studies from the SUN group, published in *Developmental Cell* and *MedComm*, collectively elucidated the protein homeostasis mechanism of DIRAS2 and proposed strategies to indirectly target KRAS^G12D^ through physiological negative regulators [2, 3]. These studies provide novel perspectives on targeting KRAS in cancer therapy beyond inhibitors.

KRAS mutations are prevalent in human cancers. These mutations prevent GTP hydrolysis, leading to sustained RAS activation, downstream signal transduction, and tumorigenesis. For decades, KRAS was considered “undruggable” due to its smooth structure, picomolar affinity for GTP, and the lack of binding pockets on the GTP‐bound form. This changed when the Food and Drug Administration approved sotorasib and adagrasib for the treatment of KRAS^G12C^ non‐small cell lung cancer (NSCLC). In recent years, inhibitors targeting G12C (Olomorasib, Opnurasib, Glecarisib, IBI351, Garsorasib, RMC‐6291, FMC‐376, and D3S‐001), G12D (MRTX1133, RMC‐9805), and pan‐RAS (RMC‐6239, BI‐3706674) have advanced into clinical trials [4]. Notably, IBI351 and Garsorasib were approved by the National Medical Products Administration in 2024 for the treatment of KRAS^G12C^ mutant NSCLC. However, no breakthroughs have yet been achieved for KRAS mutations other than G12C.

Proteolysis‐targeting chimeras (PROTACs), which recruit E3 ubiquitin ligases to target proteins for degradation via the ubiquitin‐proteasome pathway, have revolutionized drug development for challenging targets like KRAS. Popow et al. developed a PROTAC linking KRAS to the E3 ubiquitin ligase CRL2^VHL^ using a high‐affinity KRAS switch II pocket ligand previously disclosed in *Nature*. The molecule was optimized over three rounds. First, they replaced the oxygen in the linker with a methylene group to improve both cellular permeability and VHL affinity. Next, they substituted the amide with isoxazole to enhance the degradation efficiency of KRAS variants. Finally, they swapped isoxazole for triazole and added a hydroxymethyl group at the benzylic position of the VHL binder to raise in vivo active concentrations. The final molecule, ACBI3, degrades 13 of the 17 most common oncogenic KRAS variants, exhibits high‐affinity VHL engagement (binary complex half‐life >2000 s), and improves ternary complex stability (*K*_d_ = 6 nM). This drives potent KRAS degradation (half‐maximal degradation concentration = 3.9 nM in GP2d cells) and selective antiproliferative activity in KRAS mutant cells (half‐maximal inhibitory concentration = 478 nM), leading to significant tumor regressions in vivo. However, ACBI3 targets both mutant and wild‐type KRAS, which may pose potential risks, even though Popow et al. demonstrated that compound **4**, the precursor of ACBI3, does not affect the cell cycle or proliferation of wild‐type KRAS cell lines, and ACBI3 administration in vivo does not alter mouse body weight.

In addition to PROTAC, scientists have identified new targets by elucidating the regulatory mechanisms of the KRAS signaling pathway. Recently, the SUN group uncovered the protein homeostasis mechanism of DIRAS2, a poorly studied member of the DIRAS family. DIRAS2 exhibits low GTPase activity and primarily exists in a GTP‐bound form, functioning as a negative regulator of RAS. Specifically, Chang et al. discovered that ubiquitin‐conjugating enzyme E2 F (UBE2F), a NEDD8‐conjugating enzyme (E2) that specifically activates Cullin‐RING ligase 5 (CRL5) through a cascade reaction, is overexpressed in pancreatic ductal adenocarcinoma (PDAC) and associated with poor prognosis. Knockdown of UBE2F inhibits the growth and survival of KRAS mutant pancreatic cancer cells. Using a pancreas‐specific UBE2F conditional knockout (CKO) mouse model and 3D culture, they demonstrated that UBE2F deletion suppresses pancreatic tumorigenesis. After confirming that UBE2F positively regulates mitogen‐activated protein kinase (MAPK) signaling, they identified DIRAS2 as a target of UBE2F, showing that UBE2F depletion leads to CRL5 inactivation and DIRAS2 accumulation. Through in vivo and in vitro ubiquitination assays, they revealed that DIRAS2 is a substrate of CRL5 and is subjected to K11‐linked polyubiquitination. Using siRNA‐based screening of all known CRL5 receptor subunit proteins, they identified ankyrin repeat and SOCS box protein 11 (ASB11) as the substrate receptor for CRL5 that destabilizes DIRAS2. Furthermore, they demonstrated that DIRAS2 disrupts RAS‐RAF binding in a KRAS mutant‐dependent manner. The accumulation of DIRAS2 induced by UBE2F deletion suppresses pancreatic lesion development in UBE2F and DIRAS2 CKO mouse models. In contrast to UBE2F, higher DIRAS2 levels correlate with better survival in PDAC patients. Shortly after Chang et al. identified CRL5^ASB11^ as the E3 ligase for DIRAS2, Chen et al. identified ubiquitin‐specific protease 10 (USP10) as its deubiquitylase through affinity purification and mass spectrometry analysis. Using in vivo ubiquitination assays, they demonstrated that USP10 cooperates with CRL5^ASB11^ to regulate DIRAS2 stability and pancreatic cancer cell growth. These two studies collectively elucidate the mechanism of DIRAS2 ubiquitination and its role in the tumorigenesis of KRAS^G12D^ pancreatic cancer, offering several promising therapeutic targets for KRAS^G12D^ PDAC, including NEDD8 activating enzyme, UBE2F, sensitive to apoptosis gene (SAG)/RING Box protein 2 (RBX2) and ASB11 (Figure 1). However, several questions remain unresolved, such as why DIRAS2 selectively targets mutant KRAS over wild‐type RAS and whether other DUBs influence DIRAS2 activity.

**FIGURE 1:**
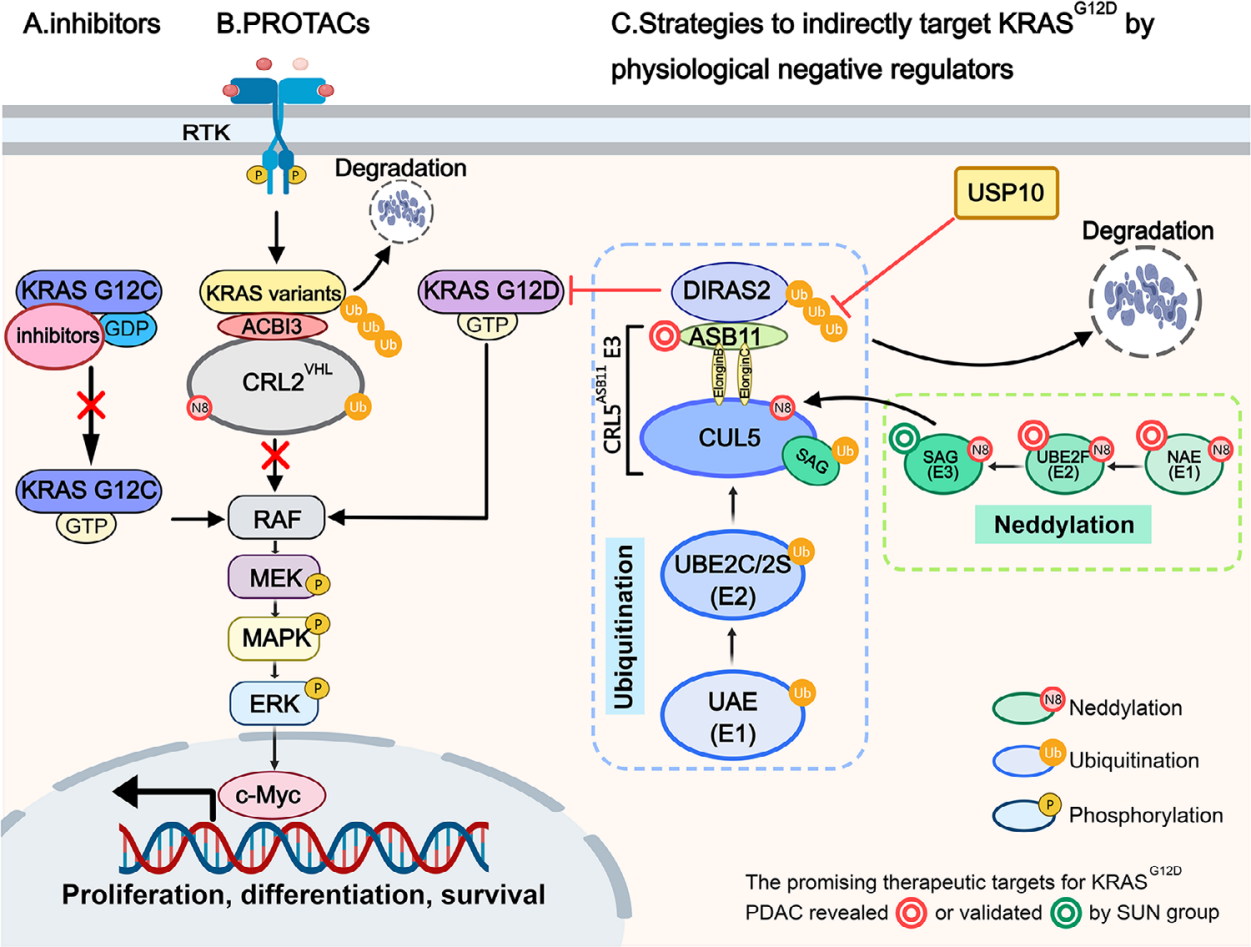
Various Strategies for Targeting KRAS. (A) Covalent inhibitors target a cysteine residue available in KRAS^G12C^. (B) Popow et al. developed a pan‐KRAS degrader designed and optimized based on a VHL:PROTAC:KRAS ternary complex to target oncogenic KRAS variants. (C) The SUN group elucidated the protein homeostasis mechanism of DIRAS2, which is regulated by its ubiquitin E3 ligase CRL5^ASB11^ and deubiquitylase USP10. They highlighted strategies to indirectly target KRAS^G12D^ pancreatic ductal adenocarcinoma (PDAC) through physiological negative regulators, offering several promising therapeutic targets. Created with MedPeer (medpeer.cn). GDP, guanosine diphosphate; GTP, guanosine triphosphate; PROTACs, proteolysis‐targeting chimeras; RTK, receptor tyrosine kinase; CRL2, Cullin‐RING ligase 2; RAF, rapidly accelerated fibrosarcoma; MEK, mitogen‐activated protein kinase kinase; MAPK, mitogen‐activated protein kinase; ERK, extracellular signal‐regulated kinase; ASB11, ankyrin repeat and SOCS box protein 11; CUL5, Cullin 5; UBE2C/2S, ubiquitin‐conjugating enzyme E2 C / ubiquitin‐conjugating enzyme E2 S; UAE, ubiquitin‐activating enzyme; USP10, ubiquitin‑specific protease 10; SAG, sensitive to apoptosis gene; UBE2F, ubiquitin‐conjugating enzyme E2 F; NAE, NEDD8 activating enzyme.

In summary, target‐driven PROTACs enable direct KRAS degradation through rational engineering, offering a unique approach to target “undruggable” proteins with high efficiency and potential to overcome drug resistance. However, they face challenges such as high hydrophobicity, poor cellular permeability, and limited tumor‐targeting ability, often requiring iterative optimization cycles, resulting in significant time, financial, and labor costs. The recently reported quantum‐classical generative model demonstrates a notable advantage in experimentally validated hits over classical models for KRAS inhibitor discovery [5]. This improved performance likely arises from quantum effects, such as superposition and entanglement. A broader application of this approach could significantly accelerate PROTAC optimization.

Unlike the well‐established inhibitors or the rapidly advancing PROTACs targeting KRAS, studies from the SUN group present a novel insight: indirectly and selectively targeting KRAS mutants by physiological negative regulators, such as DIRAS2. This approach involves more uncertainties, and its clinical translation may be prolonged due to complexity. However, it is this very uncertainty that drives a deeper understanding of KRAS regulatory mechanisms, which could even lead to breakthroughs similar to the identification of the switch II pocket, potentially overcoming the long‐standing challenges in targeting KRAS.

## Author Contributions

C.C. wrote the manuscript and prepared the figure. Z.P. provided valuable discussion. L.Z. revised and finalized the manuscript. All authors have read and approved the final manuscript.

## Ethics Statement

The authors have nothing to report.

## Conflicts of Interest

The authors declare no conflict of interest.

## Data Availability

Not applicable.

## References

[1] J. Popow , W. Farnaby , A. Gollner , et al., “Targeting Cancer with Small‐molecule Pan‐KRAS Degraders,” Science 385, no. 6715 (2024): 1338–1347. 39298590 10.1126/science.adm8684

[2] Y. Chang , Q. Chen , H. Li , et al., “The UBE2F‐CRL5(ASB11)‐DIRAS2 Axis Is an Oncogene and Tumor Suppressor Cascade in Pancreatic Cancer Cells,” Developmental Cell 59, no. 10 (2024): 1317–1332.e5. 38574733 10.1016/j.devcel.2024.03.018

[3] Q. Chen , X. Xiong , and Y. Sun , “USP10 deubiquitylates and Stabilizes DIRAS2 to Suppress the Growth of Pancreatic Cancer Cells,” MedComm 5, no. 10 (2024): e751. 39314885 10.1002/mco2.751 PMC11417426

[4] A. Singhal , B. T. Li , and E. M. O'Reilly , “Targeting KRAS in Cancer,” Nature Medicine 30, no. 4 (2024): 969–983. 10.1038/s41591-024-02903-0 PMC11845254 38637634

[5] M. Ghazi Vakili , C. Gorgulla , J. Snider , et al., “Quantum‐computing‐enhanced Algorithm Unveils Potential KRAS Inhibitors,” Nature BiotechnologyPublished online January 22, 2025. 10.1038/s41587-024-02526-3 PMC12700792 39843581

